# What–where–when memory and encoding strategies in healthy aging

**DOI:** 10.1101/lm.040840.115

**Published:** 2016-03

**Authors:** Lucy G. Cheke

**Affiliations:** Department of Psychology, University of Cambridge, Cambridge CB23EB, United Kingdom

## Abstract

Older adults exhibit disproportionate impairments in memory for item-associations. These impairments may stem from an inability to self-initiate deep encoding strategies. The present study investigates this using the “treasure-hunt task”; a what–where–when style episodic memory test that requires individuals to “hide” items around complex scenes. This task separately assesses memory for item, location, and temporal order, as well as bound what–where–when information. The results suggest that older adults are able to ameliorate integration memory deficits by using self-initiated encoding strategies when these are externally located and therefore place reduced demands on working memory and attentional resources.

In everyday life, it is often only when remembered elements of information are integrated into a single memory that they are functionally useful. You may know that you have met someone before, but if you do not remember the context—“where” and “when” you met them—you cannot be said to know “who they are.” Normal aging is thought to affect memory for these various types of information differently, and to have a particular impact on memory integration (Spencer and Raz 1995; Kessels et al. 2007; Blachstein et al. 2012). There are two main theories as to the source of these deficits. In the “associative deficit hypothesis” Naveh-Benjamin (2000) argues older adults are less able to create and retrieve links between single units of information (Chalfonte and Johnson 1996; Naveh-Benjamin 2000; Naveh-Benjamin et al. 2007). In contrast, the “hyperbinding hypothesis” (Campbell et al. 2010, 2014) suggests that older adults may be unable to down-regulate attention to irrelevant information, instead dispersing their attention across other information in the study environment that is spatially (e.g., other things in the scene) or temporally (e.g., the previous trial) close to target items. As such the correct associations are encoded, but associations are also encoded between distractor items or irrelevant environmental features, making retrieval of the target information more difficult. Both theories suggest that these impairments may be exacerbated by a failure to adopt appropriate encoding strategies. There is considerable evidence that reduced working memory resources among older adults restrict the formation of deep encoding operations, resulting in a failure to carry out “self-initiated” encoding strategies (Salthouse and Babcock 1991; Salthouse 1996; Craik and Rose 2012). However, older adults are able to benefit from encoding strategies if instructed to engage in them (Logan et al. 2002; Morcom et al. 2003).

Classical neuropsychological tools used to assess episodic memory in aging often fail to encompass the complexity of memory as it is experienced in everyday life (Piolino et al. 2009). Furthermore, episodic memory is generally assessed with verbal tasks, while most everyday memories concern visual and action information. The present study uses a what–where–when-style memory test (the treasure-hunt task) to investigate age effects on memory for self-generated temporal-spatial events. What–where–when (WWW) features are considered to be definitive of episodic memory (Tulving and Donaldson 1972) and have been extensively used to assess episodic memory behaviorally in nonhuman animals (e.g., Clayton and Dickinson 1998; Babb and Crystal 2006). Recent studies in humans have shown that WWW memories are reliably reported as “remembered” rather than “known” (Holland and Smulders 2011; Easton et al. 2012; Cheke and Clayton 2013) and are correlated with, but distinct from, free recall performance (Cheke and Clayton 2013, 2015; Mazurek et al. 2015). However, evidence for aging effects using WWW is mixed (Plancher et al. 2010, 2012; Mazurek et al. 2015).

The treasure-hunt task further differs from other episodic memory tests because the encoding period allows individuals to “hide” items themselves (Cheke et al. 2015). This is significant because agentic involvement in the encoding of items aids recollection (Plancher et al. 2012) and gets closer to memory encoding in everyday life (one does not passively observe the location of one's keys, but actively places them). Furthermore, choosing hiding locations allows participants to hide strategically. Unlike the internal strategies commonly required in verbal memory tests (such as item-categorization), these “hiding” strategies are external, and may be more similar to everyday life-encoding strategies (one does not place keys randomly then rehearse the location, one places them somewhere memorable). This might be considered in terms of changing the “environment” so as to support successful encoding. Such a strategy requires self-initiation, but arguably has a reduced working memory load compared with internal encoding strategies.

A recent neuroimaging study of young adults using a version of the treasure-hunt task found that integrated WWW memory, but not memory for the individual elements, elicited activation in the left hippocampus and angular gyrus (LG Cheke, H Bonnici, NS Clayton, JS Simons, in prep.). Both of these areas have been previously associated with memory integration (Sack 2009; Staresina and Davachi 2009; Shimamura 2011; Seghier 2013) and are known to display structural and functional changes in aging (Raz 2000; Maguire and Frith 2003; Daselaar et al. 2006; Ally et al. 2008; Giorgio et al. 2010; Rugg and Vilberg 2013). Significant activity was also seen during encoding in the right dorsolateral prefrontal cortex (RDLPFC) and activity in this area during retrieval was correlated with WWW and temporal memory performance. The RDLPFC is associated with retrieval monitoring (McDonough et al. 2013), and is consistently under-recruited by older adults during episodic memory retrieval (Cabeza et al. 1997; Grady et al. 1999; Rypma and D'Esposito 2000; Rajah and D'Esposito 2005). Furthermore, unlike in younger adults, when older adults do activate the RDLPFC it does not aid task-performance (Madden et al. 1999; Rypma and D'Esposito 2000; Cabeza et al. 2002). Given this evidence, age-related impairment might be predicted in performance on the treasure-hunt task, and particularly on integrated WWW and temporal memory. Both the associative deficit and the hyperbinding hypotheses would support such a prediction and would further predict that older adults would be less likely to engage in encoding strategies than younger adults, but that those that did use such strategies would show improved performance.

In this experiment, younger (*N* = 18, 8 male, aged 19–29 (m = 21.89, sd = 2.35)) and older (*N* = 23, 13 male, aged 60–77 (m = 66.78, sd = 4.69)) adults were recruited via posters and internet advertisement. All older adults scored above 26 on the Montreal Cognitive Assessment (range 26–40, m = 28.54, SD = 3.6), suggesting no MCI was present (Liew et al. 2014). There was no significant difference between the older and younger adults in years of education (*t*_(37)_ = 0.688, *P* = 0.496) and older adults outscored younger adults on the Shipley Institute of Living Vocabulary Scale (SILVS; *t*_(37)_ = 5.022, *P* < 0.001), which was used as a measure of crystalized IQ. All participants reported no history of mental illness and all had a BMI < 30. This study was approved by the Cambridge Psychology Research Ethics committee.

Participants completed a demographic information form, the SILVS, and a training task. They then undertook four sessions of the treasure-hunt task. The treasure-hunt task is a computer-based episodic memory test created using Psychopy (Peirce 2008) that contains five phases: Encoding, WWW, where, what, and when (see Fig. 1) presented in a fixed order. During encoding, participants were instructed to “hide” food items around a complex scene. Each item was hidden within a given scene twice, across two short (∼5 min) consecutive hiding periods (labeled “day 1” and “day 2”). Within each encoding phase, participants hid objects in two different scenes successively (such that the order was: scene 1, day 1; scene 1, day 2; scene 2, day 1; scene 2, day 2). The WWW retrieval phase occurred immediately after encoding, however because memory for scene 1 was always assessed first, the encoding of scene 2 occurred during the retention interval for scene 1 and retrieval of scene 1 occurred during the retention interval for scene 2, meaning that the retention interval was around 5 min. During the WWW retrieval phase, participants moved each item around the scene just as they had during encoding, but with the instruction “place the item in the same place you hid it on day X.” Thus they were required to indicate the location (“where”) they had hidden that item (“what”) in that scene on that “day” (“when”). This was followed by the “where” retrieval phase during which participants observed a series of “Xs” in specific locations within the scenes, half of which were old and half novel, for 5 sec. After each, they were asked “Did you hide something in that location?” The participants were then shown a series of food-items half of which were old and half novel, and asked “Did you hide this item?” (“what” retrieval period). Finally, subjects were shown two old items and asked “Which of these did you hide first?” (“when” retrieval period). Here, participants were tested on the order of items within as well as between scenes (e.g., the last item from scene 1 appeared before the first item of scene 2). While each item appeared on both “day 1” and “day 2” in each scene, the participants were asked to consider when they “first” hid that item. There were four different sessions of these tasks which were conducted in a random order, counterbalanced across individuals, however, due to loss of data, only data from two sessions are included here. In these sessions four items were hidden in each scene on each day. Thus over two sessions, participants hid a total of 16 items across 32 hiding events.

**Figure 1.**
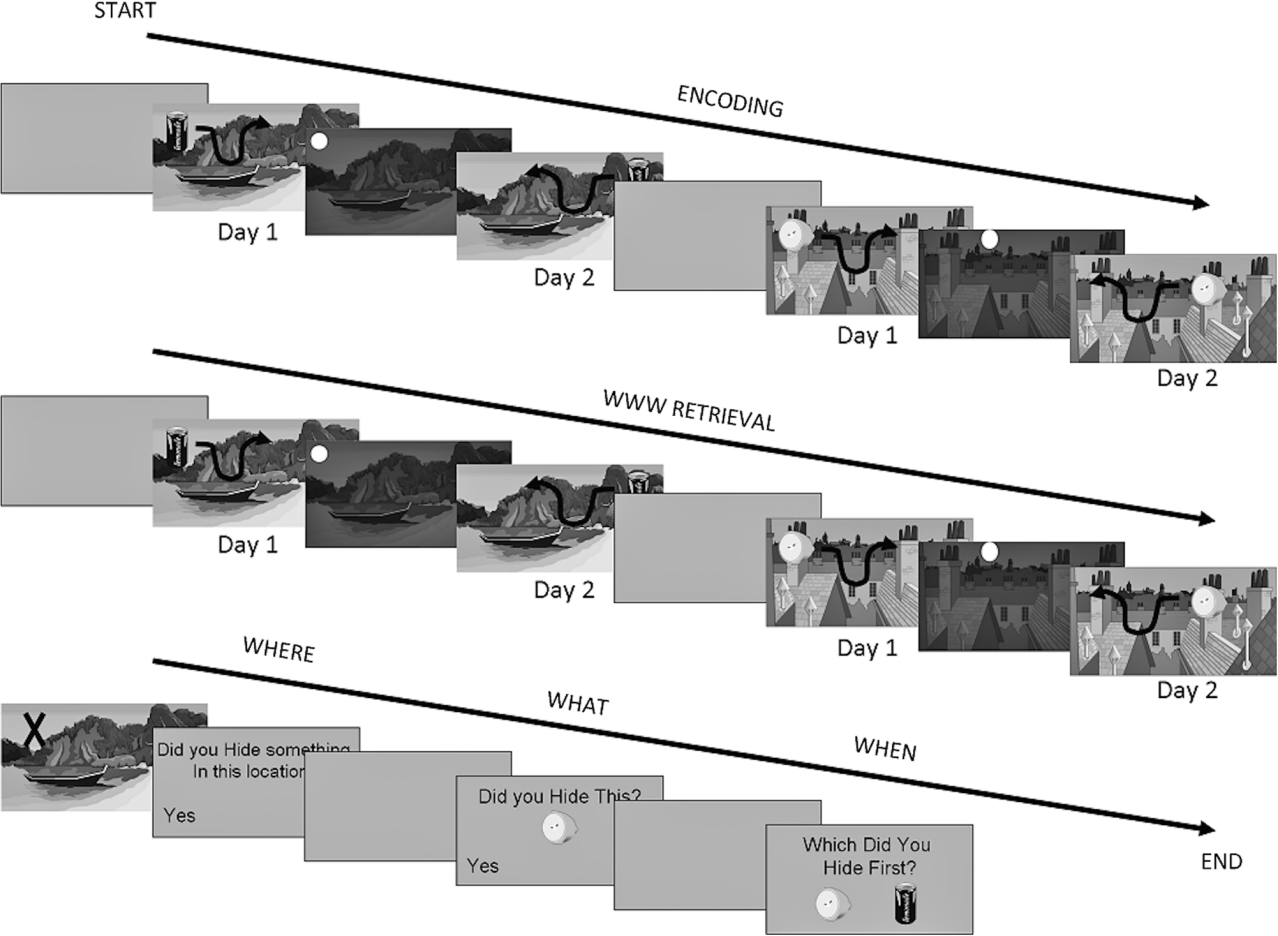
Schematic of the memory test. Participants moved items around and “hid” them in two scenes across two “days” (“encoding”). Black arrow indicates movement of item. Participants were then asked to indicate in the same manner where they hidden each food on each day (“WWW retrieval”). They were then given the “where” and “what” recognition tests, followed by “when” order discrimination test.

Accuracy on the “WWW” task was calculated as proportion of trials where participants indicated the precisely correct location of a particular item hidden on a particular day. Accuracy on the “where” and “what” tasks was computed by calculating d′ from proportion of correctly identified “old” items/locations against false alarms. Accuracy for the “when” task was computed by calculating d′ from proportion of correct against incorrect answers (Macmillan and Creelman 1990).

Participants’ hiding patterns were coded for evidence of strategies emphasizing different features. Participants were considered to be using a “what” strategy if they linked items of the same identity, for example, by hiding them near one another or consistently on the same type of landmark. A “where” strategy was evidenced by placing items on clearly defined landmarks, and a “when” strategy by evidence of a consistent system to separate items from the two hiding periods, for example, hiding “day 1” items on the left and “day 2” items on the right. A “ what–where–when” strategy was identified if all three of these criteria were met (see Fig. 2). For all four varieties of strategy, marks were awarded per pair of items, such that it was possible to have, for example, 0.75 of a WWW strategy if three pairs met all three criteria, but the fourth pair did not. Scores and strategies across the two analyzed sessions were averaged into a single score for each task or strategy type (WWW, what, where, when). Analysis was conducted using independent samples *t*-tests, multivariate ANOVA, structural equation modeling and Pearson's correlation.

**Figure 2.**
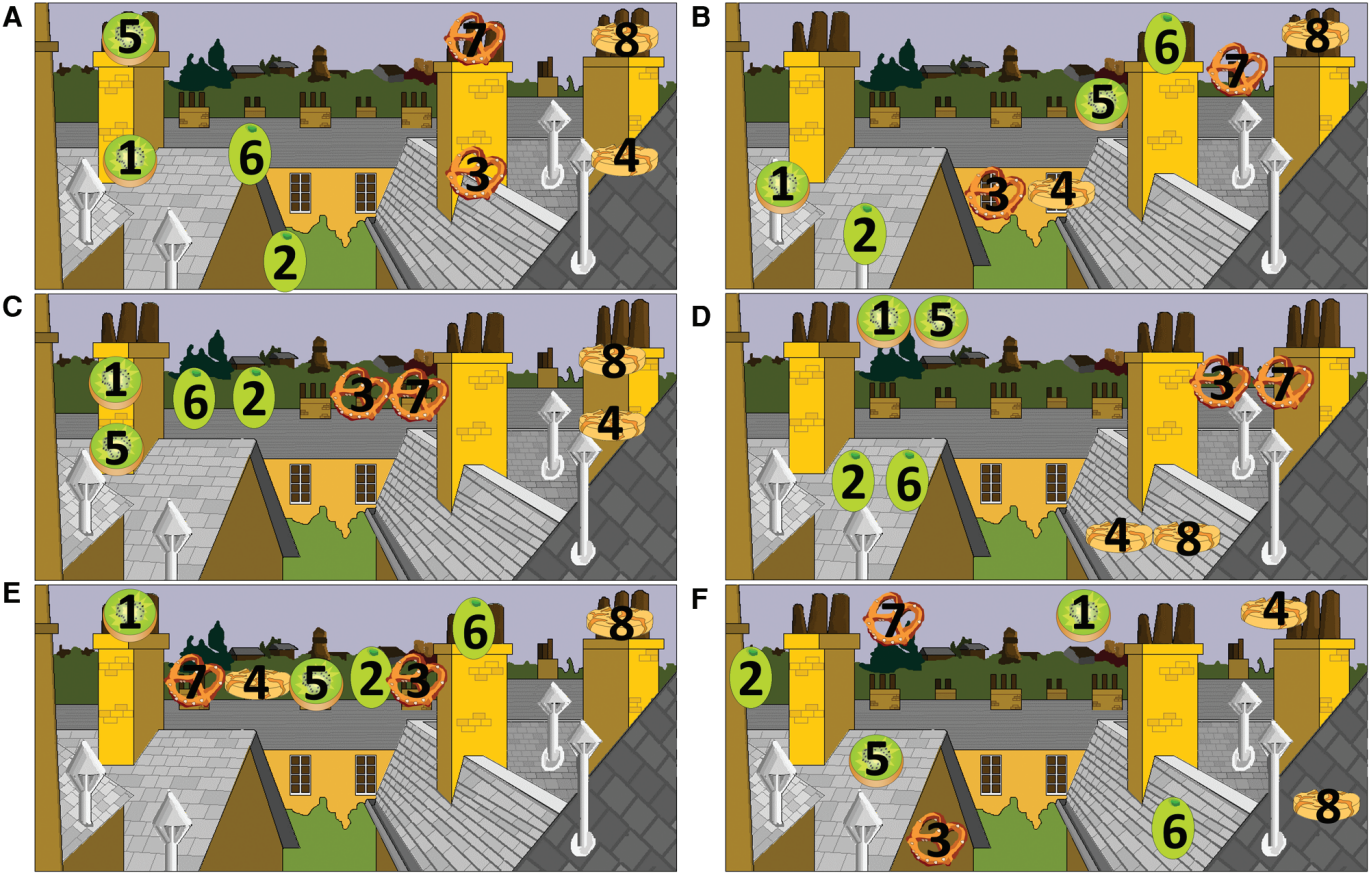
Examples of different hiding strategies. Number 1–4 indicate items from day 1 while numbers 5–8 indicate items from day 2. (*A*) Example of a what–where–when strategy: There is a clear link between items of the same identity, all the items from day 1 are underneath all the items from day 2 and each item is on a clear landmark. (*B*) Example of a where–when strategy. All items are on clear landmarks and all items from day 1 are on the left and all items from day 2 are on the right, but no link between items of the same identity. (*C*) Example of a what–where strategy: Clear link between items of the same identity, all items on a clear landmark, but no consistent placing of items from the same day. (*D*) Example of a what–when strategy: Clear link between items of the same identity, all items from day 1 on the left of items from day 2, but no evidence of hiding on a clear landmark. (*E*) Example of a where strategy. All items on a clear landmark, but no clear link between items of the same identity or from the same day. (*F*) Example with no clear strategy.

Younger adults significantly outperformed older adults on the integrated what–where–when task (*t*_(39)_ = 4.112, *P* < 0.001) and on the “when” task (*t*_(39)_ = 4.625, *P* < 0.001), but not on the “what” (*t*_(39)_ = 1.269, *P* = 0.212) or “where” (*t*_(39)_ = 0.647, *P* = 0.522) tasks. These results suggest that older adults struggled particularly with temporal memory and with integrating spatial, object, and temporal order memory (Fig. 3). Effect sizes for WWW and “when” were large (*d* = 1.3, and *d* = 1.43, respectively) but small for “what” and “where” (what: *d* = 0.41, where: *d* = 0.20) suggesting this pattern of performance did not result from a lack of power in some tests. Given that temporal information is a component part of WWW memory, structural equation modeling was conducted to investigate whether the impact of age on what–where–when performance could be dissociated from that of age on “when.” A model constrained such that age affected “when” and WWW performance independently found a significant impact of age on both memory performances (*P* < 0.001). However, when the model was amended to allow “when” performance to influence WWW performance, this association was significant (*P* < 0.001) and the direct association between age and WWW dropped below significance (*P* = 0.185).

**Figure 3.**
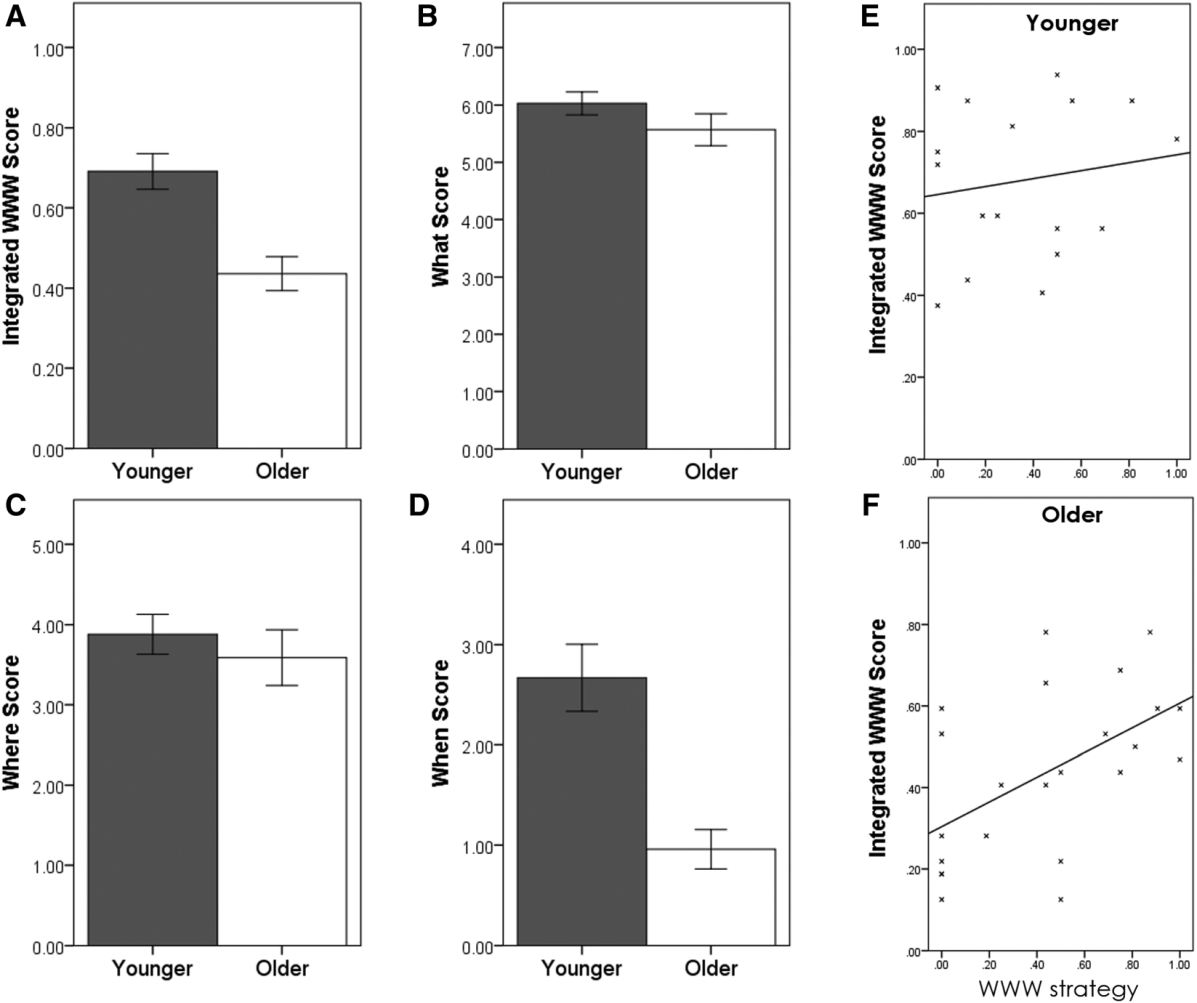
Performance on the four tests in older and younger adults (*A*–*D*) and correlation between strategy use and performance (*E*,*F*). (*A*) Proportion of what–where–when combinations correctly recalled. (*B*) *d*′ scores on the object recognition “what” test. (*C*) *d*′ scores on the spatial recognition “where” test. (*D*) *d*′ scores on the forced-choice “when” test. (*E*) Correlation between WWW strategy use and performance on the integrated WWW task in younger and (*F*) older adults.

There was no significant difference in the use of strategies between older and younger adults (what: *t*_(39)_ = −0.908, *P* = 0.369; where: *t*_(38)_ = −1.354, *P* = 0.184; when: *t*_(38)_ = −0.580, *P* = 0.565; WWW: *t*_(38)_ = −0.765, *P* = 0.449). Focusing on the key “WWW” strategies, the effect of strategizing on memory score differed significantly between the age groups (Univariate GLM age × strategy interaction: *F*_(2,37)_ = 4.410, *P* = 0.019). Figure 3, panels E and F, show that in the older group, there was a significant correlation between WWW strategizing and WWW score (*r*_(23)_ = 0.540, *P* = 0.008), while there was no such correlation in the younger group (*r*_(17)_ = 0.158, *P* = 0.545). The pattern was the same for the “where” score (older: *r*_(23)_ = 0.511, *P* = 0.013; younger: *r*_(17)_ = 0.213, *P* = 0.411), and similar though not significant for the “what” (older: *r*_(23)_ = 0.387, *P* = 0.068; younger: *r*_(17)_ = −0.174, *P* = 0.504), and “when” scores (older: *r*_(23)_ = 0.359, *P* = 0.092; younger: *r*_(17)_ = 0.238, *P* = 0.357).

These data support previous findings that integration is a primary memory deficit in older adults (Naveh-Benjamin 2000; Naveh-Benjamin et al. 2004; Campbell et al. 2010) and that older adults also struggle with temporal memory (Parkin et al. 1995; Cabeza et al. 2000). In the current study, however, whether the latter is due to a difficulty with temporal memory or integration per se, or with integrating object and temporal order is unclear, since the effect of age on what–where–when performance was mediated by that of age on “when” (essentially what–when). Given that these tasks assess slightly different types of “when” (absolute order as compared with first versus second occasion) this may suggest that the problem may lie in integrating temporal information with object information more generally. The lack of an age-related deficit in object recognition is also in line with previous literature (Craik and Byrd 1982; Grady et al. 1999; Gutchess et al. 2005; Craik and Schloerscheidt 2011; Mazurek et al. 2015).

It is notable that there was no significant difference between the older and younger adults in their tendency to use hiding strategies, but that the use of such strategies specifically benefitted the older adults. Much of the literature concerning encoding strategies in older adults has suggested that this group is less able to “self-initiate” deep encoding strategies, but benefit when they are provided with such strategies (Salthouse and Babcock 1991; Salthouse 1996; Logan et al. 2002; Morcom et al. 2003; Craik and Rose 2012). Here, unlike in most other studies, the participants were able to “externally” organize the to-be-remembered material to make it more memorable, but because they were not given any instructions, any strategies that were produced were self-initiated. External strategies can be thought of as self-generated environmental support, which has been shown to aid encoding and recall in older adults (Craik and Rose 2012). These results suggest that older adults may be able to successfully self-initiate encoding strategies if those strategies consist of an “external” manipulation of information rather than an internal change in information processing. If older adults are able to cement a strategy in an external environment—by essentially creating environmental support for themselves—then this may reduce the working memory demands and allow successful strategy implementation, leading to improved retrieval.

One caveat of this version of the treasure-hunt task is that the WWW, what, where, and when elements differed in the retrieval support provided. Some tests (e.g., what, where) required only recognition, whereas others provided fewer retrieval cues. The WWW task in particular made significant retrieval demands. Given that age-related deficits were limited to conditions with less retrieval support, general deficits in recollection could also contribute to the pattern of performance, in addition to deficits in forming/retrieving specific associations. Such difficulties have been previously demonstrated in older adults (e.g., Craik 1986). Future studies should thus use later versions of the treasure-hunt task (e.g., LG Cheke, H Bonnici, NS Clayton, JS Simons, in prep.) that control for retrieval support across the different tests.

Deficits in episodic memory early in the aging process may signal the likelihood of developing dementia later on (Bäckman et al. 2001). Given this, it is important to be able to accurately measure and monitor episodic memory ability in older adults. Here, older adults were found to be impaired on temporal and associative WWW memory, but not item or spatial memory. In contrast to previous findings, older adults were not less likely to self-initiate encoding strategies, but were more likely to benefit from them if they did so. This novel finding is possibly due to the facility to cement these encoding strategies in the external environment, rather than initiating and maintaining them internally as with traditional strategic encoding. The results presented here highlight the potential value of the treasure-hunt task in exploring the underlying sources of age-related episodic memory deficit, as well as revealing those abilities that may remain intact with age.
